# A Distance-Based Kernel Association Test Based on the Generalized Linear Mixed Model for Correlated Microbiome Studies

**DOI:** 10.3389/fgene.2019.00458

**Published:** 2019-05-16

**Authors:** Hyunwook Koh, Yutong Li, Xiang Zhan, Jun Chen, Ni Zhao

**Affiliations:** ^1^Department of Biostatistics, Johns Hopkins Bloomberg School of Public Health, Baltimore, MD, United States; ^2^School of Physics, Peking University, Beijing, China; ^3^Department of Public Health Sciences, Pennsylvania State University, Hershey, PA, United States; ^4^Department of Health Sciences Research, Mayo Clinic, Rochester, MN, United States

**Keywords:** microbiome association studies, correlated microbiome studies, longitudinal microbiome studies, community-level association analysis, distance-based association analysis, adaptive association analysis

## Abstract

Researchers have increasingly employed family-based or longitudinal study designs to survey the roles of the human microbiota on diverse host traits of interest (e. g., health/disease status, medical intervention, behavioral/environmental factor). Such study designs are useful to properly control for potential confounders or the sensitive changes in microbial composition and host traits. However, downstream data analysis is challenging because the measurements within clusters (e.g., families, subjects including repeated measures) tend to be correlated so that statistical methods based on the independence assumption cannot be used. For the correlated microbiome studies, a distance-based kernel association test based on the linear mixed model, namely, correlated sequence kernel association test (cSKAT), has recently been introduced. cSKAT models the microbial community using an ecological distance (e.g., Jaccard/Bray-Curtis dissimilarity, unique fraction distance), and then tests its association with a host trait. Similar to prior distance-based kernel association tests (e.g., microbiome regression-based kernel association test), the use of ecological distances gives a high power to cSKAT. However, cSKAT is limited to handling Gaussian traits [e.g., body mass index (BMI)] and a single chosen distance measure at a time. The power of cSKAT differs a lot by which distance measure is used. However, choosing an optimal distance measure is challenging because of the unknown nature of the true association. Here, we introduce a distance-based kernel association test based on the generalized linear mixed model (GLMM), namely, GLMM-MiRKAT, to handle diverse types of traits, such as Gaussian (e.g., BMI), Binomial (e.g., disease status, treatment/placebo) or Poisson (e.g., number of tumors/treatments) traits. We further propose a data-driven adaptive test of GLMM-MiRKAT, namely, aGLMM-MiRKAT, so as to avoid the need to choose the optimal distance measure. Our extensive simulations demonstrate that aGLMM-MiRKAT is robustly powerful while correctly controlling type I error rates. We apply aGLMM-MiRKAT to real familial and longitudinal microbiome data, where we discover significant disparity in microbial community composition by BMI status and the frequency of antibiotic use. In summary, aGLMM-MiRKAT is a useful analytical tool with its broad applicability to diverse types of traits, robust power and valid statistical inference.

## Introduction

The recent surge in next-generation sequencing technologies has dramatically advanced the human microbiome studies by enabling generic characterization of the microbes in the human body (Hamady and Knight, 2009; Caporaso et al., 2010; Thomas et al., 2012). As the sequencing technology evolves, researchers are able to obtain more accurate metagenomic information with lower cost at a faster speed. Various types of metagenomic information can be obtained by the sequencing platforms, such as microbial abundances and functional/metabolic expressions (Mallick et al., 2017). In this study, we focus on the data for the microbial abundance and phylogenetic information of the surrogate microbial species, known as, operational taxonomic units (OTUs). Furthermore, we focus on the microbiome association studies which test the disparity in microbial community (e.g., bacterial kingdom) composition by a host trait of interest (e.g., health/disease status, clinical intervention, behavioral/environmental factor) (Li, 2015). For example, recent studies have found disparity in microbial community composition for a variety of health/disease status [e.g., obesity (Arslan, 2014), type I diabetes (Zhang et al., 2018a), type II diabetes (Qin et al., 2012), human immunodeficiency virus (Bandera et al., 2018), inflammatory bowel disease (Knights et al., 2013; Borren et al., 2018), and cancers (Zitvogel et al., 2015)], medical interventions [e.g., administration of antibiotics (Zhang et al., 2018a)], and behavioral/environmental factors [e.g., diet, residence, smoking and birth mode (Charlson et al., 2010; Liu et al., 2017)].

Notably, researchers have increasingly employed family-based (Goodrich et al., 2014; Schloss et al., 2014) or longitudinal study designs (Yang et al., 2017; Zhang et al., 2018a). Such study designs are advantageous in properly controlling for potential confounders or the sensitive changes in microbial composition and host traits. That is, because family members share similar environmental/genetic factors (refer that monozygotic twins even have the same genetic background), the use of family controls can efficiently rule out some potential confounding factors. Moreover, because microbial composition and host traits can vary by time, repeated measurements over a lengthy follow-up period can ensure more reliable analysis outcomes. Examples for such correlated microbiome studies include the familial (Goodrich et al., 2014) and longitudinal (Zhang et al., 2018a) studies, the data of which we use for our real data applications (see Real data applications). Briefly, Goodrich et al. (2014) have collected stool samples from families with twins in the United Kingdom to assess the relationship between obesity and gut microbiota. Zhang et al. (2018a) longitudinally collected fecal, cecal, and ileal samples from non-obese diabetic mice to evaluate whether the intestinal microbiota altered by early-life antibiotic exposure affects maturation of innate immunity. The downstream data analysis for such studies is challenging because the measurements within clusters (e.g., families, subjects including repeated measures) tend to be correlated. We need to properly model the within-cluster correlation structure for valid statistical inferences. Besides, the unique features of the microbiome data (e.g., high-dimensionality, sparsity, and phylogenetic structure) need to be properly accounted for.

However, most of the current microbial community-level association tests [e.g., PERMANOVA (Anderson, 2001; McArdle and Anderson, 2001; Tang et al., 2016), MiRKAT (Zhao et al., 2015), MiSPU (Wu et al., 2016), OMiAT (Koh et al., 2017), aMiAD (Koh, 2018)] assume independent samples. Hence, they cannot be used for correlated microbiome studies. Zero-inflated Beta regression model (ZIBR) (Chen and Li, 2016) and negative Binomial mixed model (NBMM) (Zhang et al., 2017, 2018b) have recently been proposed for correlated microbiome studies. However, ZIBR and NBMM test individual microbial biomarkers (e.g., OTUs, taxa), not the microbial community as a whole. Hence, they are subject to a substantial loss of power after the requisite multiple testing correction. To our best knowledge, a remarkable community-level association test for correlated microbiome studies is the correlated sequence kernel association test (cSKAT) (Zhan et al., 2018). cSKAT is based on the linear mixed model (Laird and Ware, 1982), where the inherent random effect captures the within-cluster correlation of a host trait, and models the variance covariance structure of the microbial community based on an ecological distance, such as Jaccard dissimilarity (Jaccard, 1912), Bray-Curtis dissimilarity (Bray and Curtis, 1957) or unique fraction (UniFrac) distances (Lozupone and Knight, 2005; Lozupone et al., 2007; Chen et al., 2012). The use of ecological distances, which has also been widely adopted for many prior community-level association tests (Anderson, 2001; McArdle and Anderson, 2001; Zhao et al., 2015; Tang et al., 2016; Koh et al., 2017, 2018; Plantinga et al., 2017; Zhan et al., 2017), gives cSKAT a higher power than the ones based on non-ecological distances (Zhan et al., 2018). This is because the ecological distances are well-informed by properly modeling the microbial abundance and phylogenetic information (Jaccard, 1912; Bray and Curtis, 1957; Lozupone and Knight, 2005; Lozupone et al., 2007; Chen et al., 2012).

However, cSKAT has two major limitations. First, cSKAT is based on the linear mixed model (Laird and Ware, 1982). Hence, it is limited to handling Gaussian traits [e.g., body mass index (BMI)]. However, in practice, investigators can be interested in other trait types. Therefore, we introduce a distance-based kernel association test based on the generalized linear mixed model (GLMM), namely, GLMM-MiRKAT, to handle diverse types of traits, such as Gaussian (e.g., BMI), Binomial (e.g., disease status, treatment/placebo) or Poisson (e.g., number of tumors/treatments) traits. Second, cSKAT is limited to the item-by-item use of the ecological distances (i.e., the approach based on a single chosen ecological distance measure at a time). It is well-recognized in the microbiome research community that the power differs a lot by which distance measure is used, while it is also highly depending on the true underlying association pattern (Zhao et al., 2015; Koh et al., 2017, 2018). In practice, the true association pattern is usually unknown; hence, it is highly difficult to predict which distance measure performs best and choose a single optimal distance measure to use. The approach of individually testing multiple distances also requires multiple testing correction leading to a loss of power. Therefore, for a robustly high power, without the need to choose the optimal distance measure, we propose a data-driven adaptive test of GLMM-MiRKAT, namely, aGLMM-MiRKAT. aGLMM-MiRKAT robustly adapts to diverse association patterns by jointly considering multiple candidate ecological distance measures. Jaccard dissimilarity (Jaccard, 1912), Bray-Curtis dissimilarity (Bray and Curtis, 1957), UniFrac distances (Lozupone and Knight, 2005; Lozupone et al., 2007; Chen et al., 2012) are included as the candidate ecological distance measures because of their well-known features and distinguished performances (details are addressed later) (Zhao et al., 2015). Through extensive simulation experiments, we estimate robustly high power with well-controlled type I error for aGLMM-MiRKAT.

The rest of the paper is organized as follows. (1) In Materials and Methods, we address methodological details. (2) In Simulation, we address extensive simulation experiments. (3) In Real data applications, we apply aGLMM-MiRKAT to real familial and longitudinal microbiome data sets, where we test the association of the microbial community composition with BMI and the frequency of antibiotic use, while making interesting testing attempts and interpretations. (4) In Discussion, we finish with discussion and concluding remarks.

## Materials and Methods

### Notations and Models

We let *y*_*ij*_ denote a host trait of interest (e.g., health/disease status, medical intervention, behavioral/environmental factor) for the *j*-th measurement in the *i*-th cluster (*i* = 1, …, *n*, *j* = 1, …, *m*_*i*_), *z*_*ijk*_ denote the abundance level of the *k*-th OTU among *p* OTUs in the microbial community (*k* = 1, ⋯ , *p*), and *x*_*ijl*_ denote a covariate among *q* covariates (e.g., age, gender) that we want to adjust for (*l* = 1, …, *q*). We also let *N* denote the total number of measurements (i.e., *N* = $\overset{n}{\underset{i=1}{\sum }}m_{i}$), ***I***_***g***_ denote the *g*-th order identity matrix and **1**_***g***_ denote the *g* × 1 vector of ones. Throughout the paper, we use non-bold lowercase letters for scalars, bold lowercase letters for vectors, and bold uppercase letters for matrices.

To relate the microbial community composition with a host trait adjusting for covariates, we consider a generalized linear mixed model (Breslow and Clayton, 1993) (Equation 1).

(1) $$\begin{array}{l} g\left(\mu_{ij}\right)=x_{ij}^{T}\alpha +s_{ij}^{T}\upsilon_{\text{i}}+h\left(z_{ij}\right), \end{array}$$

where *g*(·) is a canonical link function (e.g., identity function for Gaussian traits, logistic function for Binomial traits, log function for Poisson traits) and μ_*ij*_ = *E*(*y*_*ij*_). **α** = ${\left(\alpha_{0},\;\ldots ,\;\alpha_{q}\right)}^{T}$ are fixed effects for the covariates *x*_*ij*_ = ${\left(1,\;x_{ij1},\;\ldots ,\;x_{ijq}\right)}^{T}$. υ_*i*_ is the random effect for the pre-specified *s*_*ij*_ to account for the within-cluster correlation in responses (i.e., conditional on υ_*i*_ and *h*(*z*_*ij*_), *y*_*ij*_ are independent with a diagonal variance-covariance matrix $\sigma_{\varepsilon }^{2}I_{m_{i}}$). For example, when *s*_*ij*_ = 1, υ_*i*_ is the random intercept which is assumed to follow a normal distribution *N*(0, $\sigma_{\gamma }^{2}$). When *s*_*ij*_ = ${\left(1,\;t_{ij}\right)}^{T}$, where *t*_*ij*_ is the time point for the *i*-th cluster and *j*-th measurement, **υ**_**i**_ = (υ_*i*1_, υ_*i*2_) is the random intercept and slope which are assumed to follow normal distributions υ_*i*1_ ~ *N*(0, $\sigma_{\gamma 1}^{2}$) and υ_*i*2_ ~*N*(0, $\sigma_{\gamma 2}^{2}$). Then, **γ**_**i**_ **≡** ${\left(s_{i1}\upsilon_{i},\;\ldots ,\;s_{im_{i}}\upsilon_{i}\right)}^{T}$follows a normal distribution with mean zero and *m*_*i*_ × *m*_*i*_ variance-covariance matrix **Σ**_*i*_. The random effect υ_*i*_ is to capture the within-cluster correlation in responses, while *h*(·) is a function which features the microbiome effect.

Here, we are particularly interested in testing ***H***_0_: *h*(*z*_*ij*_) = 0 (i.e., no association between microbial composition and a host trait adjusting for covariates) and, notably, with different specifications for *h*(*z*_*ij*_), we can characterize different association patterns between microbial composition and a host trait. One may specify *h*(*z*_*ij*_) as a fixed effect using a linear or non-linear function for the OTUs. For example, we can specify *h*(*z*_*ij*_) = ${\varphi \left(z_{ij}\right)}^{T}$**β**, where φ(·) is an element-wise transformation (e.g., identity or quadratic) function and **β** = ${\left(\beta_{1},\;\ldots ,\;\beta_{p}\right)}^{T}$ are regression coefficients for the *p* OTUs, and then test *H*_0_: **β** = **0** using a *p*-degrees of freedom test. However, because of the high-dimensional nature of the data (i.e., *p* >> *n*) and, for example, the resulting issue of low-rank matrices, testing *H*_0_: **β** = **0** with fixed effects might be challenging or even impossible. Therefore, we apply the kernel trick (Cristianini and Shawe-Taylor, 2000) and specify δ_*ij*_≡ *h*(*z*_*ij*_) = $\overset{n}{\underset{i^{\prime }\;=\;1}{\sum }}\overset{m_{i}}{\underset{j^{\prime }\;=\;1}{\sum }}\omega_{ij}\kappa \left(z_{ij},\;z_{i}^{\prime }j^{\prime }\right)$, where κ(·,·) is a positive semi-definite kernel function which measures pairwise similarities in microbial composition, *z*_*ij*_ = ${\left(z_{ij1},\;\ldots ,\;z_{ijp}\right)}^{T}$ is the *p* × 1 vector for the *p* OTUs and ω_*ij*_'s are coefficients; as such, *h*(·) lies in a reproducing kernel Hilbert space spanned by κ(·,·). Then, via the connection between kernel machine regression and mixed effect models (Liu et al., 2007), **δ** $={\;\left(\delta_{11},\ldots ,\;\delta_{1m_{1}},\;\ldots ,\;\delta_{n1},\ldots ,\;\delta_{nm_{n}}\right)}^{T}$ is assumed to follow a distribution with mean zero and variance-covariance matrix τ***K***, where **δ** is an *N* × 1 vector, τ is the unknown variance component and ***K*** is an *N* × *N* pairwise similarity matrix. Then, we can perform a variance component test for *H*_0_: τ = 0 vs. *H*_1_: τ > 0 (Lin, 1997).

To address details on the kernel matrix ***K*** and the test statistic for *H*_0_: τ = 0, we first re-write the model (Equation 1) with matrix forms for all the measurements across all the clusters (Equation 2).

(2) $$\begin{array}{l} g\left(\mu \right)=X\alpha +\gamma +\delta , \end{array}$$

where **μ** = ${\left(\mu_{11},\;\ldots ,\;\mu_{1m_{1}},\;\ldots ,\;\mu_{n1},\;\ldots ,\;\mu_{nm_{n}}\right)}^{T}$ is an *N* × 1 vector, **α** = ${\left(\alpha_{0},\;\ldots ,\;\alpha_{q}\right)}^{T}$ is an (*q*+1) × 1 vector, ***X*** = ${\left(x_{11},\;\ldots \;x_{1m_{1}},\;\ldots ,\;x_{n1},\;\ldots ,\;x_{nm_{n}}\right)}^{T}$ is an *N* × (*q*+1) matrix, **γ** = (**γ**_**1**_, …, **γ**_**n**_) is an *N* × 1 vector, and **δ** $={\;\left(\delta_{11},\ldots ,\;\delta_{1m_{1}},\;\ldots ,\;\delta_{n1},\ldots ,\;\delta_{nm_{n}}\right)}^{T}$ is an *N* × 1 vector. Again, **δ** is assumed to follow a distribution with mean zero and variance-covariance matrix τ***K***. We further assume that the two random effects **γ** and **δ** are independent as in (Lin, 1997). The kernel matrix ***K*** is an *N* × *N* pairwise similarity matrix which is converted from the use of an ecological distance (Zhao et al., 2015), such as Jaccard dissimilarity (Jaccard, 1912), Bray-Curtis dissimilarity (Bray and Curtis, 1957) or UniFrac distances (Lozupone and Knight, 2005; Lozupone et al., 2007; Chen et al., 2012), via (Equation 3).

(3) $$\begin{array}{l} K_{\left(h\right)}=-\frac{1}{2}\left(I_{N}\;-\;\frac{1_{N}1_{N}^{T}}{N}\right){\text{D}}_{\left(h\right)}^{2}\left(I_{N}\;-\;\frac{1_{N}1_{N}^{T}}{N}\right), \end{array}$$

where ***D***_(*h*)_ is the *N* × *N* pairwise distance matrix and $D_{\left(h\right)}^{2}$ is its element-wise square matrix, where *h* is an index for a chosen measure among diverse ecological distances. This kernel matrix (Equation 3) externally models ecologically meaningful pairwise similarities (correlation) in microbial composition among all the measurements across all the clusters, where the block-diagonals (i.e., ***K***_(1,*m*_1_), (1, *m*_1_)_, ***K***_(*m*_1_+;1, *m*_1_+;*m*_2_ ), (*m*_1_+;1, *m*_1_+;*m*_2_)_, …, ***K***_(*N*−*m*_*n*_+;1, *N*), (*N*−*m*_*n*_+;1, *N*)_) model the within-cluster similarities while the off-diagonals model the between-cluster similarities. The extent of OTU abundance and phylogenetic information is properly modulated by different ecological distance measures (Zhao et al., 2015).

### GLMM-MiRKAT

While we will soon address the issue that the testing performance differs according to the choice of distance measure, we first introduce the variance component score statistic for a single chosen distance measure (i.e., item-by-item approach). Following (Lin, 1997), the variance component score statistic can be formulated with (Equation 4). Here, we construct the kernel matrix *K*_(*h*)_ based on an ecological distance, and all the detailed derivation procedures are referred to (Lin, 1997).

(4) $$\begin{array}{l} \frac{\partial l(\alpha ,\gamma ,\;\tau )}{\partial \tau }{|}_{\tau =0,\;\alpha ={\hat{\alpha }}_{0},\gamma ={\hat{\gamma }}_{0}}\\ =\frac{1}{2}{\left(y^{*}-X{\hat{\alpha }}_{0}\right)}^{T}{\hat{V}}_{0}^{-1}K_{\left(h\right)}{\hat{V}}_{0}^{-1}(y^{*}-X{\hat{\alpha }}_{0})+tr({\hat{V}}_{0}^{-1}K_{\left(h\right)}), \end{array}$$

where y^*^= $X{\hat{\alpha }}_{0}$ + ${\hat{\gamma }}_{0}$ + ${\hat{\Delta }}_{0}$(***y*** *-* ${\hat{\mu }}_{0}$) is the working vector and ${\hat{V}}_{0}^{-1}$ = ${\left({\hat{\Sigma }}_{0}+{\hat{W}}_{0}\right)}^{-1}$. Here, ${\hat{\Delta }}_{0}$ = *diag*($\left(g^{\prime }({\hat{\mu }}_{0})\right)$)) (i.e., ${\hat{\Delta }}_{0}$ = ***I***_***N***_, ${\hat{\Delta }}_{0}$ = *diag* $\left({\left({\hat{\mu }}_{0}(1-{\hat{\mu }}_{0})\right)}^{-1}\right)$ and ${\hat{\Delta }}_{0}$ = *diag*(${{\hat{\mu }}_{0}}^{-1}$) for Gaussian, Binomial, Poisson traits, respectively), ${\hat{\Sigma }}_{0}=$ *diag*(${\hat{\Sigma }}_{1,0},\ldots$, ${\hat{\Sigma }}_{n,0}$), and ${\hat{W}}_{0}$ is the dispersion parameter for the errors estimated as ${\hat{W}}_{0}$ = *diag*($var\left({\hat{\mu }}_{0}\right)$, …, $var\left({\hat{\mu }}_{0}\right)$) for Gaussian traits and ${\hat{W}}_{0}$ = ***I***_**N**_ for Binomial and Poisson traits, where ${\hat{\alpha }}_{0}$, ${\hat{\gamma }}_{0}$, ${\hat{\mu }}_{0}$ and ${\hat{\Sigma }}_{0}$ are estimated under the null generalized linear mixed model by the restricted maximum likelihood estimation (REML) method (Harville, 1977) and *var*(·) is the variance function. This test statistic (Equation 4) is the penalized quasi-likelihood estimating equation in Breslow and Clayton (1993) and the variance component score statistic for testing random effects in Lin (1997) under the above model specifications. This is also the unadjusted variance component score statistic proposed for cSKAT which is based on the linear mixed model for Gaussian traits (Zhan et al., 2018). Similar test statistics have also been widely used for various family-based and longitudinal studies in genetics and neuroscience (Schifano et al., 2012; Chen et al., 2013; Zhang et al., 2014; Wang et al., 2017), while assuming different variance covariance structures and/or applying different weighting schema. Since our *p*-value computation is based on a permutation approach, the *scaling* (i.e., $\frac{1}{2}$) and *additive* [i.e., *tr*(${\hat{V}}_{0}^{-1}K_{\left(h\right)}$)] terms do not change the comparative ranks of the observed and null (i.e., permuted) statistic values (see *P*-value calculation). Hence, we use a reduced-form statistic (Equation 5).

(5) $$Q_{\left(h\right)}={\left(y^{*}-X{\hat{\alpha }}_{0}\right)}^{T}{\hat{V}}_{0}^{-1}K_{\left(h\right)}{\hat{V}}_{0}^{-1}\left(y^{*}-X{\hat{\alpha }}_{0}\right)$$

### aGLMM-MiRKAT

The testing performance depends on the choice of distance measure (Zhao et al., 2015). To explain, non-phylogeny-based distances, such as Jaccard (1912) and Bray and Curtis (1957) dissimilarities, measure the disparity only in abundance, while phylogeny-based distances, such as UniFrac distances (Lozupone and Knight, 2005; Lozupone et al., 2007; Chen et al., 2012), measure the disparity both in abundance and phylogeny. Hence, non-phylogeny-based distances are well-suited when associated OTUs have disparity in abundance, while phylogeny-based distances are well-suited when they have disparity both in abundance and phylogeny. Moreover, Jaccard dissimilarity and unweighted UniFrac distance are based on incidence information (i.e., presence/absence of OTUs), while Bray-Curtis dissimilarity and weighted UniFrac distance are based on full abundance information [refer that generalized UniFrac distance modulates the intensity of abundance information between unweighted and weighted UniFrac distances by its parameter θ (Chen et al., 2012)]. Hence, Jaccard dissimilarity and unweighted UniFrac distance are well-suited when associated OTUs are rare in abundance in the sense that prevalent OTUs are likely to exist in all samples, while Bray-Curtis dissimilarity and weighted UniFrac distance are well-suited when they are rich in abundance. However, prior knowledge about the true association pattern is usually absent in reality. Hence, it is highly challenging to choose a single optimal distance measure to use. For a robustly high performance throughout various (but unknown) association scenarios, we propose aGLMM-MiRKAT which is based on the test statistic of the minimum *p*-value from multiple item-by-item GLMM-MiRKAT analyses (Equation 6).

(6) $$\begin{array}{l} T_{aGLMMMiKAT}=\underset{h\in \Gamma }{min}P_{\left(h\right)}, \end{array}$$

where *h* is an index for a distance in a set of candidate ecological distances (Γ), where Γ = {Jaccard dissimilarity, Bray-Curtis dissimilarity, Unweighted UniFrac distance, Generalized UniFrac distance (θ = 0.5), Weighted UniFrac distance}. Obviously, we do not report the genuine minimum *p*-value (i.e., *T*_*aGLMMMiKAT*_) as it is. Instead, *T*_*aGLMMMiKAT*_ (Equation 6) is the test statistic of aGLMM-MiRKAT, and we estimate the *p*-value for aGLMM-MiRKAT (*P*_*aGLMMMiKAT*_) using a permutation approach (see *P*-value calculation). Our extensive simulations reveal that aGLMM-MiRKAT maintains high power throughout all surveyed association scenarios, while the item-by-item GLMM-MiRKAT analyses are limitedly powerful only for some association scenarios. Further details are addressed in the Simulation section.

### *P*-value Calculation

We calculate the *p*-values for the item-by-item GLMM-MiRKAT tests and aGLMM-MiRKAT using a permutation approach. Our permutation approach is semi-parametric as we fit the null model *g*(${\hat{\mu }}_{0}$) = $X{\hat{\alpha }}_{0}$ + ${\hat{\gamma }}_{0}$ (Equation 2) (excluding the microbiome portion) parametrically, and then draw the empirical null distribution of the test statistic (Equations 5, 6) through permutations non-parametrically. In this way, we can estimate the *p*-values without making distributional assumptions for the microbiome portion. Moreover, we do block permutations to account for any potential mis-specified within-cluster correlation structure based on the procedures in (Winkler et al., 2015). To be specific, for the random intercept model [i.e., *r*_*ij*_ = 1 (Equation 1)], we permute (1) the whole clusters (only the exchangeable clusters which have the same number of measurements) and (2) the measurements within each cluster, simultaneously. For the random slope model [i.e., *r*_*ij*_ = ${\left(1,\;t_{ij}\right)}^{T}$ (Equation 1)], we permute only the whole clusters (the exchangeable clusters which have the same number of measurements and the same time points). The detailed procedures for our permutation approach can be found in S1. Computational algorithm.

## Results

### Simulation

#### Simulation Designs

Our simulation designs are based on prior studies (Zhao et al., 2015; Koh et al., 2017; Zhan et al., 2018), but here we conduct more extensive simulation experiments for diverse trait types with different within-cluster correlation structures. In particular, we simulated the data for Gaussian, Binomial and Poisson traits, respectively, based on the following generalized linear mixed models.

$$\begin{array}{ll} y_{ij}=0.5\times scale\left(x_{i1}+x_{ij2}\right)\\ +\beta \times scale\left(\sum_{a\in A}z_{ija}\right)+s_{ij}^{T}\upsilon_{i}+\epsilon_{ij}\\ logit\left(E\left(y_{ij}=1\right)\right)=0.5\times scale\left(x_{i1}+x_{ij2}\right)\\ + & \beta \times scale\left(\sum_{a\in A}z_{ija}\right)+s_{ij}^{T}\upsilon_{i}\\ log\left(E\left(y_{ij}\right)\right)=0.5\times scale\left(x_{i1}+x_{ij2}\right)\\ + & \beta \times scale\left(\sum_{a\in A}z_{ija}\right)+s_{ij}^{T}\upsilon_{i} \end{array}$$

In these equations, ***x***_***i*1**_ is a cluster-specific (e.g., gender) covariate generated from the Bernoulli distribution with success probability 0.5, and *x*_*ij*2_ is a non-cluster-specific (e.g., time-varying) covariate generated from 0.5 × *scale*($\underset{a\in A}{\sum }z_{ija}$) + *N*(0, 1). Note that, *x*_*ij*2_ is a confounder as it is associated with both of the microbial composition and host trait. A is a set of associated OTUs among the total *p* OTUs in the community, and ***z***_***ija***_ is the *a*-*th* OTU in A. β is a regression coefficient for the OTUs in A. *scale* is the standardization function to have mean zero and standard deviation one. υ_*i*_ is the random effect for the pre-specified *s*_*ij*_, and ε_*ij*_ are errors generated from *N*(0, 1). We investigate small (*n* = 20) and moderate (*n* = 50) numbers of clusters, respectively, while assigning two, three and four measurements, respectively, into each one third of the clusters (i.e., when *n* = 20, *m*_*i*_ = 2 for *i* = 1, …, 7, *m*_*i*_ = 4 for *i* = 8, …, 14 and *m*_*i*_ = 3 for *i* = 15, …, 20; when *n* = 50, *m*_*i*_ = 2 for *i* = 1, …, 17, *m*_*i*_ = 3 for *i* = 18, …, 34 and *m*_*i*_ = 4 for *i* = 35, …, 50). This is to mimic (possibly) unbalanced numbers of measurements across clusters. As before, we let *i* = 1, …, *n, j* = 1, …, *m*_*i*_, *k* = 1, …, *p* and *l* = 1, …, *q*. For the random effect *v*_*i*_, we generate (1) random intercepts and (2) random intercepts and slopes, respectively, as follows. For the random intercepts (i.e., *s*_*ij*_ = 1), we generate *v*_*i*_ from *N*(0, $\sigma_{\gamma }^{2}$), while setting $\sigma_{\gamma }^{2}$ = $\frac{1}{2}$, 1 and $\frac{3}{2}$, respectively, to investigate different within-cluster correlations, that is, $\rho_{j{\neq j}^{\prime }}$ = $\sigma_{\gamma }^{2}/(\sigma_{\gamma }^{2}+\sigma_{\varepsilon }^{2})$) = $\frac{1}{3}$, $\frac{1}{2}$ and $\frac{3}{5}$. For the random intercepts and slopes (i.e., ***s***_***ij***_ = (1, *j*)^*T*^), we generate *v*_*i*1_ and *v*_*i*2_ from *N*(0, $\sigma_{\gamma }^{2}$), while setting $\sigma_{\gamma }^{2}$ = $\frac{1}{2}$, 1 and $\frac{3}{2}$, respectively and *t*_*ij*_ = *j*, to investigate different within-cluster correlations, that is, $\rho_{j{\neq j}^{\prime }}$ = $\sigma_{\gamma }^{2}/(\sigma_{\gamma }^{2}+\sigma_{\varepsilon }^{2})$) = $\frac{\left(1+j^{2}\right)}{\left(j^{2}+3\right)}$, $\frac{\left(1+j^{2}\right)}{\left(j^{2}+2\right)}$ and $\frac{\left(1+j^{2}\right)}{\left(j^{2}+\;\frac{5}{3}\right)}$.

For the OTUs in the community, we first estimated proportional means and a dispersion parameter for 856 OTUs (i.e., *p* = 856) in the bacterial kingdom from the real respiratory-tract microbiome data (Charlson et al., 2010). Then, OTU counts for each measurement per cluster (i.e., *Z*_*ij*_ for *i* = 1, …, *n, j* = 1, …, *m*_*i*_) were generated from the Dirichlet-multinomial distribution (Mosimann, 1962) with the pre-specified parameter values of the estimated proportional means and dispersion. The total reads for each measurement were set to be 10,000. To reflect possible within-cluster relatedness among microbial communities, we updated the second and third measurements of microbial community using a random perturbation function: *Z*_*ij*_ = $\frac{1}{2}$ (*Z*_*i*(*j*−1)_ + *Z*_*ij*_) for *j* = 2, …, *m*_*i*_.

To estimate empirical type I error rates, we set β = 0. To estimate statistical powers, we set β = 1, while selecting a set of associated OTUs (A) by four different association scenarios as in Koh et al. (2017, 2018) and Koh (2018) (1) 50 random OTUs among the OTUs in lower half of abundance, (2) 50 random OTUs, (3) 50 random OTUs among the OTUs in upper half of abundance, and (4) OTUs in a cluster among 10 clusters partitioned by the partition around medoids (PAM) algorithm (Reynolds et al., 2006) based on OTUs' cophenetic distances (Sneath et al., 1975), respectively. The first three scenarios mimic the situations when associated OTUs are rare, medium and abundant, respectively, while the fourth scenario mimics the situation when they are close in phylogeny. For the fourth scenario, we randomized the selection of an associated cluster among the 10 clusters to avoid arbitrary cluster selection. To estimate empirical type I error rates, we conducted 30,000 replicates for each combination of the model, sample size and correlation structure. To estimate statistical powers, we conducted 10,000 replicates for each combination of the model, sample size, correlation structure and association scenario.

##### Model fitting

We fit the random intercept model (i.e., *s*_*ij*_ = 1) when the random intercepts are generated, and we fit the random slope model (i.e., ***s***_***ij***_ = (1, *j*)^*T*^) when the random intercepts and slopes are generated, while including the two covariates and all the 856 OTUs in the community.

#### Simulation Outcomes

##### Type I error

We estimate well-controlled empirical type I error rates at the significance level of 0.05 for any item-by-item GLMM-MiRKAT or aGLMM-MiRKAT test, for any type of traits (i.e., Gaussian, Binomial and Poisson traits), for both small (*n* = 20) and moderate (*n* = 50) numbers of clusters, for any imposed within-cluster correlation, and for both random intercept (Table 1) and slope models (Table 2). However, we estimate inflated empirical type I error rates (>0.05) for the prior microbial community-level association tests, OMiRKAT (Zhao et al., 2015), aMiSPU (Wu et al., 2016), OMiAT (Koh et al., 2017), and aMiAD (Koh, 2018) (Table 3). This is because these tests treat all the measurements across all the clusters as independent samples in an exaggerated manner. We also observe in general that the higher the within-cluster correlation, the greater the type I error inflation (Table 3), as explained by the higher the within-cluster correlation, the smaller the effective sample size.

**Table 1:** Estimated type I error rates at the significance level of 5% for GLMM-MiRKAT/aGLMM-MiRKAT based on the random intercept model with Gaussian, Binomial or Poisson responses (Unit: %).

	***n*** **=** **20**			***n*** **=** **50**		
**$\rho_{j{\neq j}^{\prime }}$**	**L**	**M**	**H**	**L**	**M**	**H**
**Gaussian**						
*K*_*J*_	5.06	4.89	5.12	5.08	5.06	4.98
*K*_*BC*_	4.78	4.80	4.85	4.83	4.86	4.73
*K*_*U*_	5.07	4.96	5.04	5.19	5.05	5.06
*K*_0.5_	5.03	4.83	4.94	5.15	4.95	4.74
*K*_*W*_	4.97	5.00	4.91	4.75	4.73	4.54
adaptive	4.89	4.74	4.74	4.92	4.79	4.73
**Binomial**						
*K*_*J*_	5.08	4.93	4.91	5.00	5.13	4.88
*K*_*BC*_	4.98	4.95	4.92	5.29	5.00	4.96
*K*_*U*_	5.09	5.04	5.00	5.08	5.19	4.74
*K*_0.5_	5.05	4.88	4.89	5.03	5.13	5.12
*K*_*W*_	4.92	4.89	5.04	5.11	4.90	5.11
adaptive	4.87	4.90	4.89	5.06	4.99	4.92
**Poisson**						
*K*_*J*_	4.98	4.93	5.11	4.95	5.17	5.06
*K*_*BC*_	5.04	5.03	4.69	5.01	4.95	5.03
*K*_*U*_	5.07	4.85	5.16	4.95	5.17	5.06
*K*_0.5_	5.10	4.92	4.85	4.97	4.95	5.02
*K*_*W*_	5.11	4.87	4.64	5.03	5.09	4.90
adaptive	4.96	4.91	4.83	4.95	5.00	5.07

*K_J_: Jaccard dissimilarity; K_BC_: Bray-Curtis dissimilarity; K_U_: Unweighted UniFrac distance; K_0.5_: Generalized UniFrac distance (θ = 0.5); K_W_: Weighted UniFrac distance; adaptive: adaptive GLMM-MiRKAT (aGLMM-MiRKAT). L: low within-cluster correlation ($\rho_{j{\neq j}^{\prime }}$ = $\frac{1}{3}$); M: medium within-cluster correlation ($\rho_{j{\neq j}^{\prime }}$ = $\frac{1}{2}$); H: high within-cluster correlation ($\rho_{j{\neq j}^{\prime }}\;=$$\frac{3}{5}$)*.

**Table 2:** Estimated type I error rates at the significance level of 5% for GLMM-MiRKAT/aGLMM-MiRKAT based on the random slope model with Gaussian, Binomial or Poisson responses (Unit: %).

	***n*** **=** **20**			***n*** **=** **50**		
**$\rho_{j{\neq j}^{\prime }}$**	**L**	**M**	**H**	**L**	**M**	**H**
**Gaussian**						
*K*_*J*_	5.10	4.96	5.12	4.87	4.98	5.04
*K*_*BC*_	5.11	4.89	4.97	5.10	4.88	5.03
*K*_*U*_	5.03	4.95	5.13	5.03	5.03	5.10
*K*_0.5_	5.07	4.91	4.90	4.89	4.91	5.09
*K*_*W*_	4.96	4.95	4.87	4.83	5.03	5.01
adaptive	4.97	4.94	5.01	4.94	4.86	5.04
**Binomial**						
*K*_*J*_	5.08	4.80	5.01	5.09	5.02	4.83
*K*_*BC*_	4.93	4.94	5.1	4.89	5.02	4.88
*K*_*U*_	5.04	4.99	5.04	5.07	5.40	4.83
*K*_0.5_	5.02	4.97	4.84	5.00	5.08	4.96
*K*_*W*_	4.89	5.07	5.02	4.96	5.08	4.85
adaptive	4.99	4.94	4.85	4.86	5.11	4.82
**Poisson**						
*K*_*J*_	5.01	4.98	4.76	4.93	5.10	4.90
*K*_*BC*_	5.16	4.76	5.02	5.03	5.03	5.02
*K*_*U*_	4.90	5.06	4.92	5.09	5.19	4.93
*K*_0.5_	5.14	4.87	5.10	4.85	4.88	5.10
*K*_*W*_	5.12	4.82	5.28	4.86	5.06	5.18
adaptive	5.05	4.70	4.88	5.00	4.94	4.78

*K_J_: Jaccard dissimilarity; K_BC_: Bray-Curtis dissimilarity; K_U_: Unweighted UniFrac distance; K_0.5_: Generalized UniFrac distance (θ = 0.5); K_W_: Weighted UniFrac distance; adaptive: adaptive GLMM-MiRKAT (aGLMM-MiRKAT). L: low within-cluster correlation ($\rho_{j{\neq j}^{\prime }}$ = $\frac{\left(1+j^{2}\right)}{\left(j^{2}+3\right)}$); M: medium within-cluster correlation ($\rho_{j{\neq j}^{\prime }}$ = $\frac{\left(1+j^{2}\right)}{\left(j^{2}+2\right)}$); H: high within-cluster correlation ($\rho_{j{\neq j}^{\prime }}$ = $\frac{\left(1+j^{2}\right)}{\left(j^{2}+\;\frac{5}{3}\right)}$)*.

**Table 3:** Estimated type I error rates at the significance level of 5% for the prior microbial community-level association tests, OMiRKAT, aMiSPU, OMiAT, and aMiAD, for the clustered microbiome data (Unit: %).

**Random intercepts**						
	***n*** **=** **20**			***n*** **=** **50**		
$\rho_{j{\neq j}^{\prime }}$	**L**	**M**	**H**	**L**	**M**	**H**
**Gaussian**						
OMiRKAT	24.36	79.89	97.44	37.98	96.61	99.96
aMiSPU	14.64	52.5	80.78	20.47	75.69	95.65
OMiAT	22.13	79.27	97.77	40.63	98.65	99.97
aMiAD	5.70	6.79	8.22	6.11	7.39	8.82
**Binomial**						
OMiRKAT	7.12	20.19	41.40	9.35	30.02	62.19
aMiSPU	6.17	12.32	24.13	6.88	16.18	34.86
OMiAT	6.87	18.54	39.62	9.09	33.68	71.1
aMiAD	5.41	5.71	6.31	5.64	5.98	6.62
**Random intercepts and slopes**						
**Gaussian**						
OMiRKAT	81.86	99.27	99.89	97.53	99.92	99.94
aMiSPU	72.20	96.42	98.58	92.87	99.88	99.98
OMiAT	81.31	99.41	99.91	98.70	99.93	99.97
aMiAD	8.59	10.68	11.57	8.51	10.24	10.58
**Binomial**						
OMiRKAT	23.98	63.69	84.53	36.73	86.82	97.98
aMiSPU	15.87	42.33	62.83	21.83	63.68	84.62
OMiAT	22.64	63.08	85.10	40.63	93.27	99.49
aMiAD	6.15	7.30	8.35	6.20	7.45	8.24

*L: low within-cluster correlation ($\rho_{j{\neq j}^{\prime }}$ = $\frac{1}{3}$ for the random intercepts, $\rho_{j{\neq j}^{\prime }}$ = $\frac{\left(1+j^{2}\right)}{\left(j^{2}+3\right)}$ for the random intercepts and slopes); M: medium within-cluster correlation ($\rho_{j{\neq j}^{\prime }}$ = $\frac{1}{2}$ for the random intercepts, $\rho_{j{\neq j}^{\prime }}$ = $\frac{\left(1+j^{2}\right)}{\left(j^{2}+2\right)}$ for the random intercepts and slopes); H: high within-cluster correlation ($\rho_{j{\neq j}^{\prime }}$ = $\frac{3}{5}$ for the random intercepts, $\rho_{j{\neq j}^{\prime }}$ = $\frac{\left(1+j^{2}\right)}{\left(j^{2}+\frac{5}{3}\right)}$ for the random intercepts and slopes)*.

##### Power

**Figure 1:**
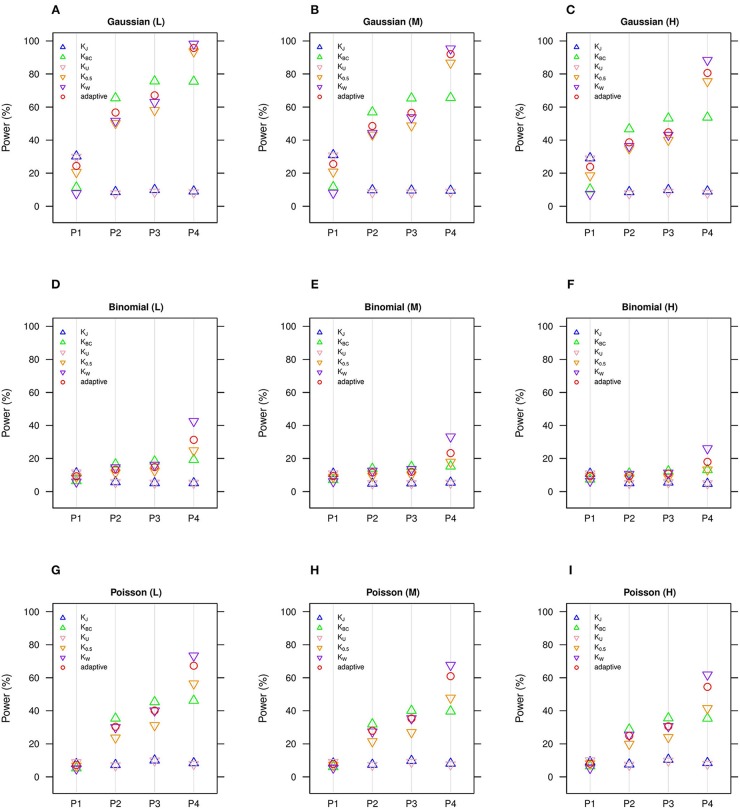
Estimated statistical powers for GLMM-MiRKAT/aGLMM-MiRKAT based on the random intercept model with Gaussian, Binomial or Poisson responses (*n* = 50) (Unit: %). L: low within-cluster correlation ($\rho_{j{\neq j}^{\prime }}$ = $\frac{1}{3}$); M: medium within-cluster correlation ($\rho_{j{\neq j}^{\prime }}$ = $\frac{1}{2}$); H: high within-cluster correlation ($\rho_{j{\neq j}^{\prime }}\;=$ $\frac{3}{5}$).*K*_*J*_: Jaccard dissimilarity; *K*_*BC*_: Bray-Curtis dissimilarity; *K*_*U*_: Unweighted UniFrac distance; *K*_0.5_: Generalized UniFrac distance (θ = 0.5); *K*_*W*_: Weighted UniFrac distance; *adaptive*: adaptive GLMM-MiRKAT (aGLMM-MiRKAT). P1, P2, P3, and P4 represent the four different association scenarios: P1. A = {50 random OTUs in lower half of abundance}; P2. A = {50 random OTUs}; P3. A = {50 random OTUs in upper half of abundance}; P4. A = {A random cluster among 10 clusters partitioned by PAM}. **(A)** Gaussian (L); **(B)** Gaussian (M); **(C)** Gaussian (H); **(D)** Binomial (L); **(E)** Binomial (M); **(F)** Binomial (H); **(G)**. Poisson (L); **(H)** Poisson (M); **(I)**. Poisson (H).

**Figure 2:**
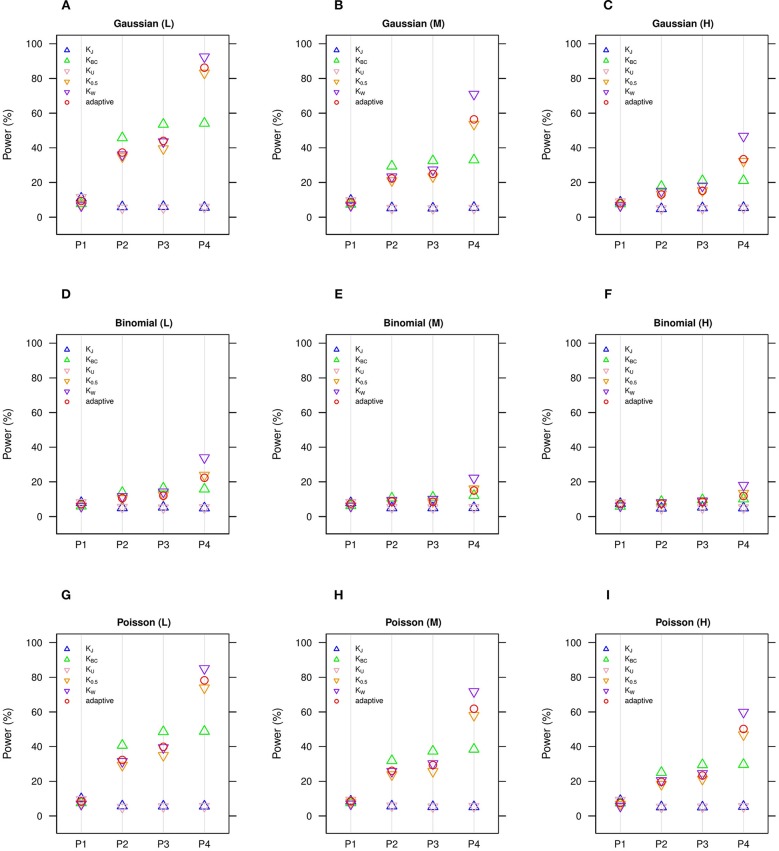
Estimated statistical powers for GLMM-MiRKAT/aGLMM-MiRKAT based on the random slope model with Gaussian, Binomial or Poisson responses (*n* = 50) (Unit: %). L: low within-cluster correlation ($\rho_{j{\neq j}^{\prime }}$ = $\frac{1}{3}$); M: medium within-cluster correlation ($\rho_{j{\neq j}^{\prime }}$ = $\frac{1}{2}$); H: high within-cluster correlation ($\rho_{j{\neq j}^{\prime }}\;=$ $\frac{3}{5}$). *K*_*J*_: Jaccard dissimilarity; *K*_*BC*_: Bray-Curtis dissimilarity; *K*_*U*_: Unweighted UniFrac distance; *K*_0.5_: Generalized UniFrac distance (θ = 0.5); *K*_*W*_: Weighted UniFrac distance; *adaptive*: adaptive GLMM-MiRKAT (aGLMM-MiRKAT). P1, P2, P3, and P4 represent the four different association scenarios: P1. A = {50 random OTUs in lower half of abundance}; P2. A = {50 random OTUs}; P3. A = {50 random OTUs in upper half of abundance}; P4. A = {A random cluster among 10 clusters partitioned by PAM}. **(A)** Gaussian (L); **(B)** Gaussian (M); **(C)** Gaussian (H); **(D)** Binomial (L); **(E)** Binomial (M); **(F)** Binomial (H); **(G)** Poisson (L); **(H)** Poisson (M); **(I)** Poisson (H).

We estimate in general that the moderate number of clusters (*n* = 50) (Figures 1, 2) is more powerful than the small number of clusters (*n* = 20) (Figures S1, S2), yet we observe the same comparative powers among different GLMM-MiRKAT analyses for the small (*n* = 20) and moderate (*n* = 50) number of clusters. Thus, to save space, the power outcomes for the small (*n* = 20) number of clusters are placed in (Figures S1,S2).

We estimate in general that the Gaussian models (Figures 1A–C, 2A–C) are more powerful than the Binomial (Figures 1D–F, 2D–F) and Poisson (Figures 1G–I, 2G–I) models, where the Binomial models are the least powerful. This is because the continuous traits are better informed than the discrete traits, but not because our methods better suit the Gaussian models. We also observe in general that the higher the within-cluster correlation, the lower the power (i.e., Figures 1A,D,G, 2A,D,G > Figures 1B,E,H, 2B,E,H > Figures 1C,F,I, 2C,F,I), as explained by the higher the within-cluster correlation, the smaller the effective sample size. We observe similar comparative powers among different GLMM-MiRKAT analyses across Gaussian, Binomial and Poisson models for both of the random intercept (Figure 1) and slope (Figure 2) models. We address the detailed description on the comparative powers below.

GLMM-MiRKAT using Jaccard dissimilarity or unweighted UniFrac distance is more powerful in the first scenario when associated OTUs are rare in abundance (Figures 1, 2: P1), while GLMM-MiRKAT using Bray-Curtis dissimilarity or weighted UniFrac distance is relatively more powerful in the second and third scenarios when associated OTUs are mid-abundant and abundant (Figures 1, 2: P2-P3), as expected by their distinct weighting schema. GLMM-MiRKAT using weighted UniFrac distance or generalized UniFrac distance is more powerful in the fourth scenario when associated OTUs are close in phylogeny (Figures 1, 2: P4), where GLMM-MiRKAT using Jaccard dissimilarity or Bray-Curtis dissimilarity is less powerful (Figures 1, 2: P4), as expected by their use or non-use of phylogenetic information. Notably, none of the item-by-item GLMM-MiRKAT analyses are consistently powerful throughout all different association scenarios (i.e., they are powerful for some scenarios to which they are well-suited, but they are under-powered for the other scenarios to which they are not well-suited) (Figures 1, 2). On the contrary, we estimate that the adaptive test of GLMM-MiRKAT, aGLMM-MiRKAT, is robustly powerful (closely reaching the highest power among the item-by-item GLMM-MiRKAT analyses) throughout all different association scenarios (Figures 1,2).

**Figure 3:**
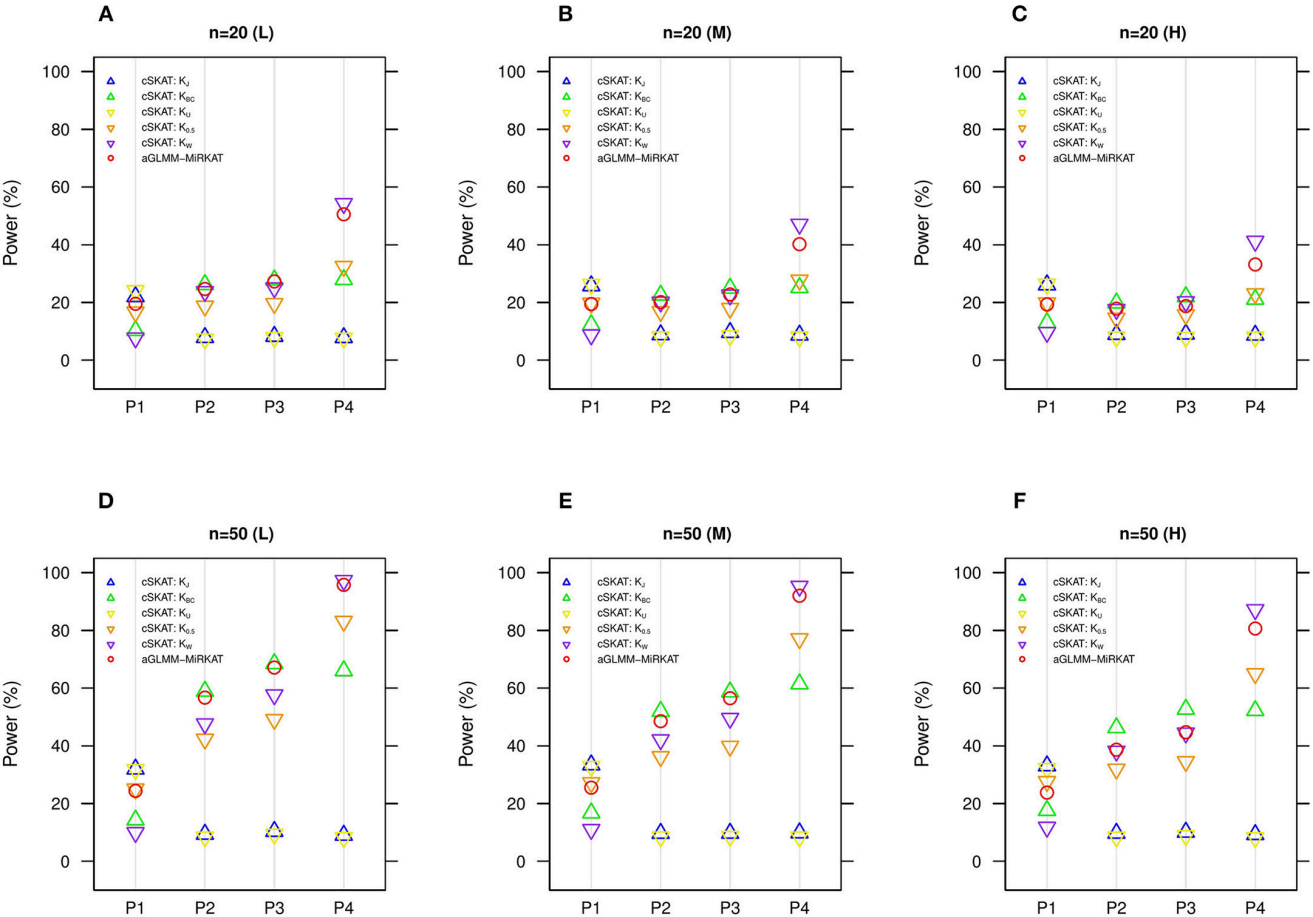
Estimated statistical powers for the item-by-item cSKAT tests and aGLMM-MiRKAT based on the random intercept model with Gaussian responses (*n* = 50) (Unit: %). L: low within-cluster correlation ($\rho_{j{\neq j}^{\prime }}$ = $\frac{1}{3}$); M: medium within-cluster correlation ($\rho_{j{\neq j}^{\prime }}$ = $\frac{1}{2}$); H: high within-cluster correlation ($\rho_{j{\neq j}^{\prime }}\;=$ $\frac{3}{5}$).*K*_*J*_:cSKAT for Jaccard dissimilarity; *K*_*BC*_:cSKAT for Bray-Curtis dissimilarity; *K*_*U*_:cSKAT for Unweighted UniFrac distance; *K*_0.5_: cSKAT for Generalized UniFrac distance (θ = 0.5); *K*_*W*_: cSKAT for Weighted UniFrac distance; *adaptive*: adaptive GLMM-MiRKAT (aGLMM-MiRKAT). P1, P2, P3, and P4 represent the four different association scenarios: P1. A = {50 random OTUs in lower half of abundance}; P2. A = {50 random OTUs}; P3. A = {50 random OTUs in upper half of abundance}; P4. A = {A random cluster among 10 clusters partitioned by PAM}. **(A)** *n* = 20 (L); **(B)** *n* = 20 (M); **(C)** *n* = 20 (H); **(D)** *n* = 50 (L); **(E)** *n* = 50 (M); **(F)** *n* = 50 (H).

We additionally compare aGLMM-MiRKAT with the item-by-item cSKAT analyses for the random intercept Gaussian models as cSKAT can handle only the Gaussian traits based on the random intercept model (Zhan et al., 2018). Similar to the previous item-by-item GLMM-MiRKAT analysis outcomes, none of the item-by-item cSKAT analyses are consistently powerful throughout all different association scenarios (i.e., they are powerful for some scenarios to which they are well-suited, but they are under-powered for the other scenarios to which they are not well-suited) (Figure 3). Here again, we observe that aGLMM-MiRKAT maintains a high power throughout all different scenarios (Figure 3).

### Real Data Applications

#### A Family-Based Study on the Association Between Obesity and Gut Microbiota

Goodrich et al. (2014) have collected fecal samples from the United Kingdom twin population to study the roles of host genetics on gut microbiome, while addressing a breadth of associations between obesity indices and gut microbiota. Here, we analyze a small portion the original data to evaluate the association between BMI and microbial community composition. The raw sequence data are publicly available in the European Bioinformatics Institute (EBI) repository (Assess codes: ERP006339 and ERP006342). We processed them using the QIIME pipeline (Caporaso et al., 2010) with open reference-based OTU picking by targeting the V4 region of the 16S ribosomal RNA (rRNA) gene, and quantified OTUs at the 97% sequence similarity level and constructed a phylogenetic tree. Among the total of 1,024 measurements from 536 families, we focused on monozygotic twins. After excluding measurements with low sequencing depth (i.e., < 10,000 total reads), 311 measurements from 145 families were included in our analysis. The data originally include 7,365 OTUs, but we removed OTUs with average relative abundance < 10^−5^, and then the data were rarefied to control unequal library sizes (Weiss et al., 2017); as such, 2,128 OTUs were included in our analysis.

**Figure 4:**
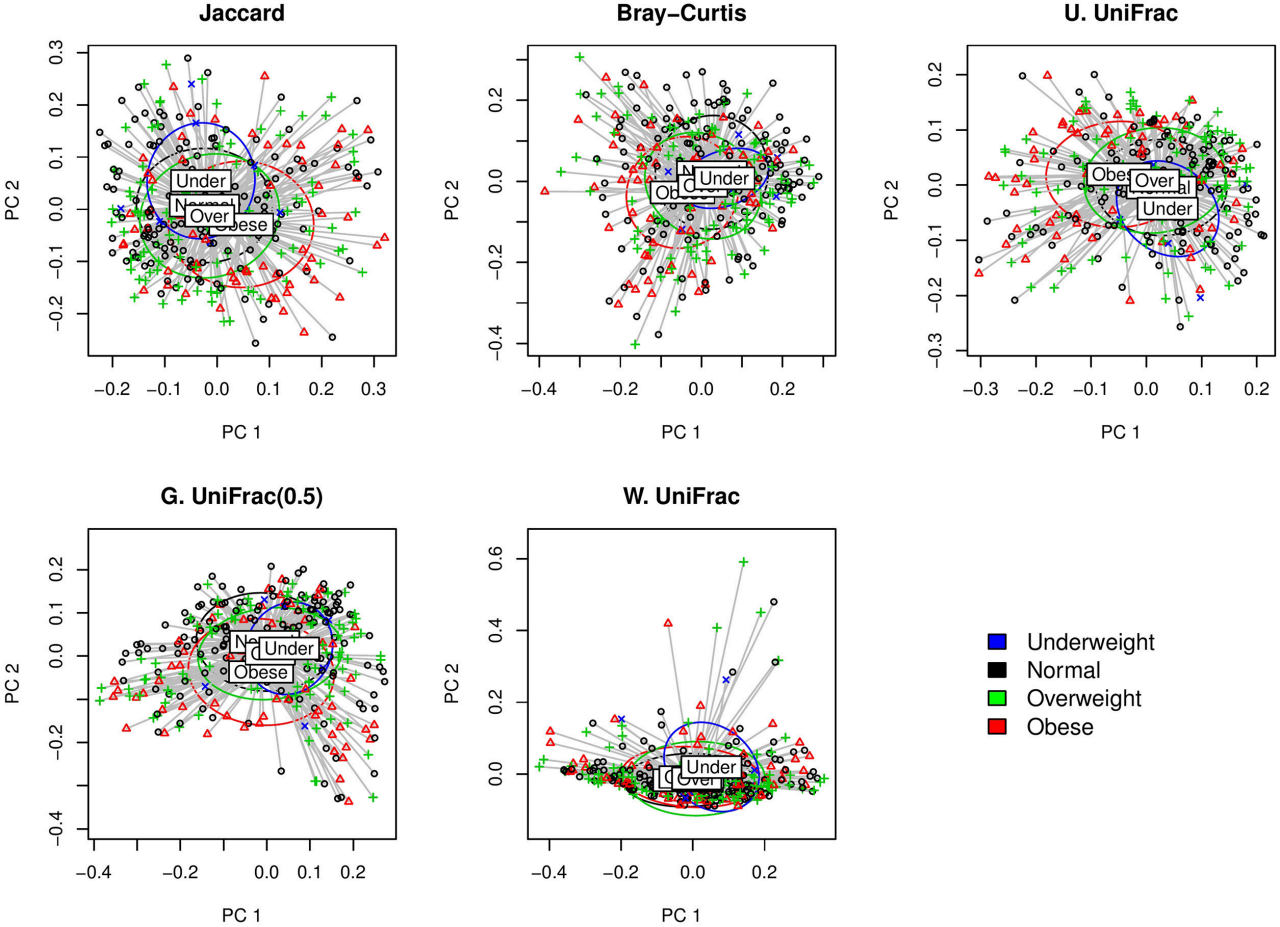
The two-dimensional PCoA plots depicting the microbial profiles among BMI categories (i.e., Under: BMI < 18.5; Normal: 18.5 ≤ BMI < 25; Over: 25 ≤ BMI < 30; Obese: 30 ≤ BMI). Jaccard: Jaccard dissimilarity; Bray-Curtis: Bray-Curtis dissimilarity; U. UniFrac: unweighted UniFrac distance; G. UniFrac: generalized UniFrac distance (θ = 0.5); W. UniFrac: weighted UniFrac distance.

We first visually check with principle coordinate analysis (PCoA) plots based on each distance measure to see if there is any disparity in microbial composition by BMI categories [i.e., under-weighted: BMI ($\frac{kg}{m^{2}}$) < 18.5; normal: 18.5 ≤ BMI ($\frac{kg}{m^{2}}$) < 25; over-weighted: 25 ≤ BMI ($\frac{kg}{m^{2}}$) < 30; obese: 30 ≤ BMI ($\frac{kg}{m^{2}}$)] (Figure 4). It is not very clear in the visual inspection if there is any significant separation by BMI categories, and we observe the smallest separation based on weighted UniFrac distance (Figure 4).

We fitted GLMM-MiRKAT with random intercepts for BMI in continuous scale (Gaussian traits) adjusting for age. GLMM-MiRKAT using Jaccard dissimilarity (*p*-value: < 0.001), Bray-Curtis dissimilarity (*p*-value: < 0.001), unweighted UniFrac distance (*p*-value: < 0.001) or generalized UniFrac distance (θ = 0.5) (*p*-value: 0.005) estimates significant association between BMI and microbial composition, while GLMM-MiRKAT using weighted UniFrac distance (*p*-value: 0.157) does not. This matches with our visual inspection of the smallest separation for the weighted UniFrac distance (Figure 4). This also indicates that the item-by-item GLMM-MiRKAT analyses are considerably sensitive to the choice of distance measure. aGLMM-MiRKAT estimates the significant association (*p*-value: < 0.001).

For another demonstration, we fitted GLMM-MiRKAT with random intercepts for BMI in binary scale (Binomial traits) adjusting for age, comparing the normal and obese populations (i.e., 140 measurements from 85 families in the normal vs. 63 measurements from 41 families in the obese). However, we could not find any significant association by any item-by-item [i.e., Jaccard dissimilarity (*p*-value: 0.354), Bray-Curtis dissimilarity (*p*-value: 0.107), unweighted UniFrac distance (*p*-value: 0.336), generalized UniFrac distance (θ = 0.5) (*p*-value: 0.231), weighted UniFrac distance (*p*-value: 0.333)] or adaptive [i.e., aGLMM-MiRKAT (*p*-value: 0.253)] analysis. This power loss, of course, is related to the reduced sample size in the selected comparison. This may also indicate that BMI in continuous scale is better informed than BMI in binary scale, which matches with our simulation result, where the Gaussian models are more powerful than the Binomial models (Figures 1,2).

#### A Longitudinal Study on the Association Between the Frequency of Antibiotic Use and Gut Microbiota

Zhang et al. (2018a) collected fecal, cecal and ileal samples from non-obese diabetic mice for microbiome profiling studies based on a longitudinal study design to evaluate if the intestinal microbiota altered by early-life antibiotic exposure affects maturation of innate immunity. The raw sequence data are publicly available in the Qiita database (Identifier: 11242). We processed them using the QIIME pipeline (Caporaso et al., 2010) with open reference-based OTU picking by targeting the V4 region of the 16S rRNA gene, and quantified OTUs at the 97% sequence similarity level and constructed a phylogenetic tree. The original study (Zhang et al., 2018a) contains enormous amount of data for a number of sub-studies, but, for a demonstration of our proposed method, we only analyze a small portion of the data. To be specific, we focused on fecal samples to evaluate the disparity in microbial community composition by the frequency of antibiotic use (i.e., 0, 1, 2, and 3 course(s) of antibiotic use). After excluding measurements with low sequencing depth (i.e., < 10,000 total reads), 229 measurements from 87 mice were included in our analysis. The study design is longitudinal and unbalanced in that each mouse has different numbers of repeated measurements: 61 mice have three measurements, 20 mice have two measurements and 6 mice have one measurement through different time points. Among the total of 229 measurements, 120 have had no antibiotic use, 43 have had one course of antibiotic use, 26 have had two courses of antibiotic use, and 40 have had three courses of antibiotic use.

**Figure 5:**
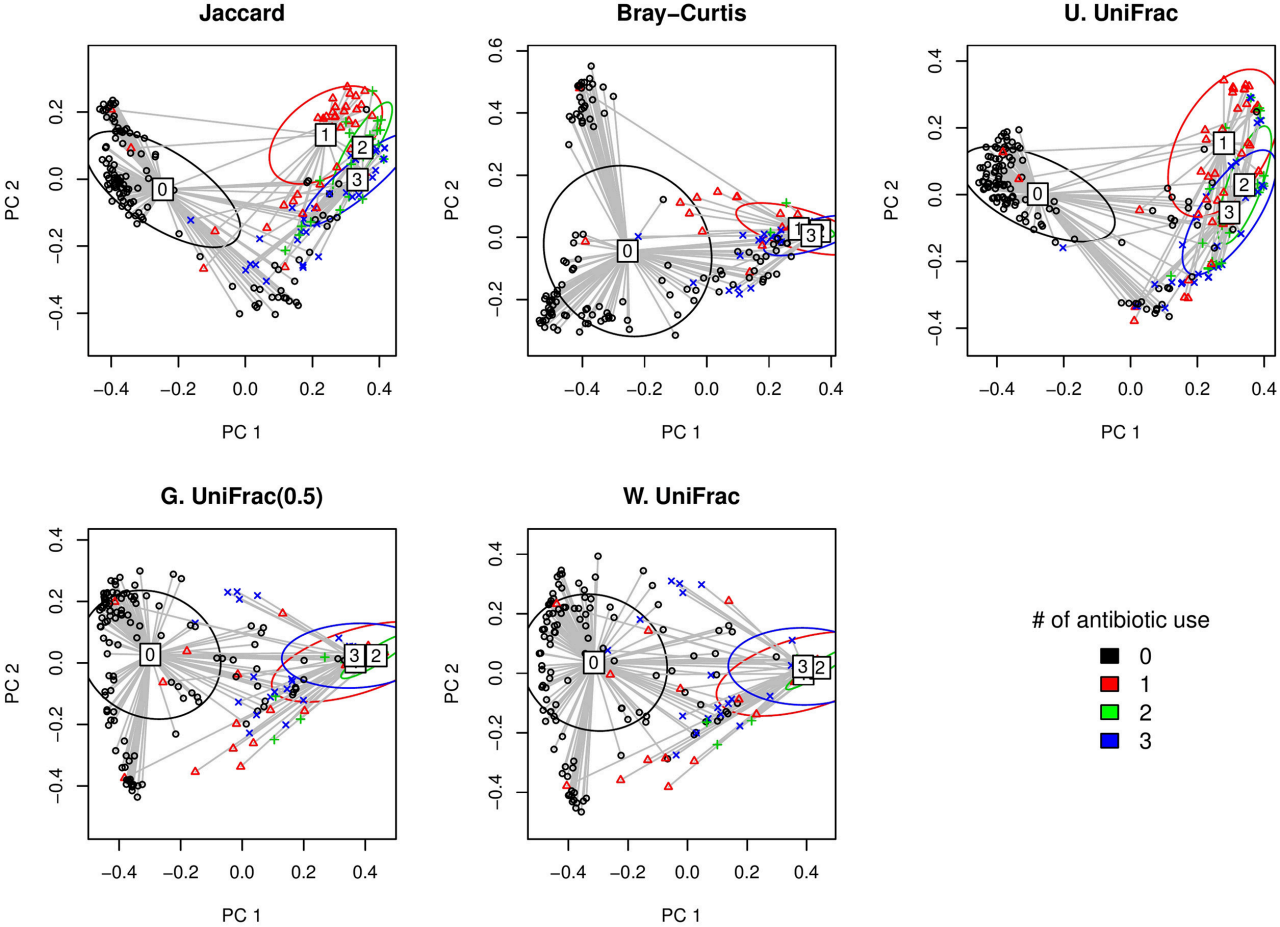
The two-dimensional PCoA plots depicting the microbial profiles among different groups defined by the number of antibiotic use (i.e., 0, 1, 2, and 3 course(s) of antibiotic use). Jaccard: Jaccard dissimilarity; Bray-Curtis: Bray-Curtis dissimilarity; U. UniFrac: unweighted UniFrac distance; G. UniFrac: generalized UniFrac distance (θ = 0.5); W. UniFrac: weighted UniFrac distance.

Here, we first visually check with the PCoA plots based on each distance measure to see if there is any disparity in microbial composition by different numbers of antibiotic use (Figure 5). We observe a very clear visual separation, especially from no antibiotic use group to at least one course of antibiotic use group, based on any distance measures (Figure 5).

We fitted GLMM-MiRKAT with random intercepts for the number of antibiotic use (Poisson traits) (i.e., 0, 1, 2, and 3 course(s) of antibiotic use) adjusting for gender. We found significant association between the number of antibiotic use and microbial composition by all the item-by-item analysis [i.e., Jaccard dissimilarity (*p*-value: < 0.001), Bray-Curtis dissimilarity (*p*-value: < 0.001), unweighted UniFrac distance (*p*-value: < 0.001), generalized UniFrac distance (θ = 0.5) (*p*-value: < 0.001), weighted UniFrac distance (*p*-value: < 0.001)]. We also found the significant association for aGLMM-MiRKAT (*p*-value: < 0.001).

## Discussion

In this paper, we introduced a distance-based kernel association test based on the generalized linear mixed model, GLMM-MiRKAT, for correlated (e.g., family-based or longitudinal) microbiome studies. GLMM-MiRKAT can relate microbial community composition with any type of host traits that are distributed as an exponential family distribution. Thus, GLMM-MiRKAT can be regarded as an extension of cSKAT (Zhan et al., 2018) to handle non-Gaussian host traits. Furthermore, we developed aGLMM-MiRKAT to incorporate multiple kernels for a robustly high power. aGLMM-MiKRAT is especially useful in practice, where there are various types of host traits, but our knowledge about the true association pattern is limited.

We calculate the *p*-values for the item-by-item GLMM-MiRKAT and aGLMM-MiRKAT using a permutation approach. The permutation approach is robust to any small or large sample size without making distributional assumptions. GLMM-MiRKAT/aGLMM-MiRKAT can be implemented for either the random intercept model or the random slope model while cSKAT is only for the random intercept model. For the random intercept model, we permute both the whole exchangeable clusters and the measurements within each cluster. We can do so because the random intercept model assumes an exchangeable (a.k.a. *compound symmetry*) within-cluster correlation structure. Therefore, for the random intercept model, our permutation approach works in any study design with either balanced or unbalanced numbers of measurements per cluster. However, for random intercept model, we permute only the whole exchangeable clusters. Therefore, for the random slope model, our permutation approach is limited to the balanced study design with a sufficient number of whole exchangeable clusters. In practice, the random intercept model has been more widely used for many prior tests (Min and Agresti, 2005; Schifano et al., 2012; Chen et al., 2013; Zhang et al., 2014; Chen and Li, 2016; Wang et al., 2017) because the random intercepts are usually sufficient to capture the within-cluster correlation structure in responses. The model selection procedures are beyond the scope of this study and we defer the details to popular longitudinal data analysis books.

Throughout this paper, we have surveyed the bacterial kingdom as the microbial community of interest because it is usually in our shared interest (bacteria make up most of the human microbiota). However, without loss of generality, the methods can be applied to any other microbial communities, such as the kingdom of yeasts, fungi or viruses, or the lower level microbial assemblages (e.g., phyla, classes) (Koh et al., 2017). We use OTUs as the sub-units consisting of the microbial community because they are often used as the surrogate microbial species. However, any other sub-units (e.g., phylum, species, genera) can be alternatively used by researchers' choice. We considered the ecological distance measures [i.e., Jaccard dissimilarity (Jaccard, 1912), Bray-Curtis dissimilarity (Bray and Curtis, 1957) or UniFrac distances (Lozupone and Knight, 2005; Lozupone et al., 2007; Chen et al., 2012)] due to their popularity in the microbiome research community. However, any other distance measures or kernel matrices can be alternatively used by researcher's choice. We also make no distinction between the 16S rRNA gene sequencing (Hamady and Knight, 2009; Caporaso et al., 2010) and the shotgun metagenomic sequencing (Thomas et al., 2012) for the use of our proposed methods.

## Author Contributions

HK, NZ, and YL developed the method. HK performed the simulation experiments and real data analyses, and developed the software package. NZ, XZ, and JC contributed to simulations and real data analyses. HK and NZ wrote the manuscript. All authors read and approved the final manuscript.

### Conflict of Interest Statement

The authors declare that the research was conducted in the absence of any commercial or financial relationships that could be construed as a potential conflict of interest.
