# Supplementary material for: A Distance-Based Kernel Association Test Based on the Generalized Linear Mixed Model for Correlated Microbiome Studies
Source: Front Genet. 2019 May 16;10:458. doi: 10.3389/fgene.2019.00458 (PMC6532659; doi:10.3389/fgene.2019.00458)

S1. Computational algorithm

1. Fit the null generalized linear mixed effect model and obtain $\hat{\boldsymbol{y}}^*$ = $X\hat{\boldsymbol{\alpha}}_{\mathbf{0}}$ + $\hat{\boldsymbol{\gamma}}_{\mathbf{0}}$ + $\hat{\Delta}_0(\boldsymbol{y}$ - $\hat{\boldsymbol{\mu}}_{\mathbf{0}})$ and $\hat{V}_0^{-1}$ = $(\hat{\Sigma}_0 + \hat{W}_0)^{-1}$. Denote the $N \times 1$ residual vector $\boldsymbol{r}$ = $\hat{\boldsymbol{y}}^* - X\hat{\boldsymbol{\alpha}}_{\mathbf{0}}$.
2. Compute the observed statistic value $Q_{(h)}$ = $\boldsymbol{r}^T\hat{V}_0^{-1}K_{(h)}\hat{V}_0^{-1}\boldsymbol{r}$ for each distance $h \in \Gamma$.
3. For the random intercept model, randomly shuffle the components of the residual vector $\boldsymbol{r}$ simultaneously i) by clusters (only the exchangeable clusters (blocks) which are matched in the number of measurements) and ii) the measurements within each cluster. For the random slope model, randomly shuffle the components of the residual vector $\boldsymbol{r}$ by clusters (only the exchangeable clusters (blocks) which are matched in the number of measurements and time points). Repeat this procedure many times (say, B times) and denote each permuted residual vector as $\boldsymbol{r}'_{(b)}$, b = 1, …, B.
4. Compute the null statistic values $Q_{(h)(b)}$ = ${\boldsymbol{r}'_{(b)}}^T\hat{V}_0^{-1}K_{(h)}\hat{V}_0^{-1}\boldsymbol{r}'_{(b)}$, b = 1, …, B, for each distance $h \in \Gamma$.
5. Compute the p-values $P_{(h)}$ = $\sum_{b=1}^{B}[I(\,Q_{(h)(b)} \geq Q_{(h)})$+1]/($B$+1) for each distance $h \in \Gamma$, where $I(\cdot)$ is an indicator function.
6. Compute the observed statistic value $T_{aGLMMMiKAT}$ = $\min_{h\in\Gamma} P_{(h)}$.
7. Compute the null statistic values $T_{aGLMMMiKAT(b)}$ = $\min_{h\in\Gamma}\{\sum_{b'\neq b}[\,I(T_{aGLMMMiKAT(b')} \geq T_{aGLMMMiKAT(b)})$+1]/$B$}, b = 1, …, B and b' = 1, …, B, where $I(\cdot)$ is an indicator function.
8. Calculate the p-value $P_{aGLMMMiKAT}$ = $\sum_{b=1}^{B}[I(\,T_{aGLMMMiKAT(b)} \leq T_{aGLMMMiKAT})$+1]/($B$+1), where $I(\cdot)$ is an indicator function.

Figure S1. Estimated statistical powers for GLMM-MiRKAT/aGLMM-MiRKAT based on the random intercept model with Gaussian, Binomial or Poisson responses (*n*=20) (Unit: %). L: low within-cluster correlation ($\rho_{j\neq j'}$ = 1/3); **M:** medium within-cluster correlation ($\rho_{j\neq j'}$ = 1/2); **H:** high within-cluster correlation ($\rho_{j\neq j'}$ = 3/5).$\boldsymbol{K_J}$: Jaccard dissimilarity; $\boldsymbol{K_{BC}}$: Bray-Curtis dissimilarity; $\boldsymbol{K_U}$: Unweighted UniFrac distance; $\boldsymbol{K_{0.5}}$: Generalized UniFrac distance (θ=0.5); $\boldsymbol{K_W}$ **:** Weighted UniFrac distance; ***adaptive*****:** adaptive GLMM-MiRKAT (aGLMM-MiRKAT). **P1**, **P2**, **P3** and **P4** represent the four different association scenarios: **P1.** $\mathcal{A}$ = {50 random OTUs in lower half of abundance}; **P2.** $\mathcal{A}$ = {50 random OTUs}; **P3.** $\mathcal{A}$ = {50 random OTUs in upper half of abundance}; **P4.** $\mathcal{A}$ = {A random cluster among 10 clusters partitioned by PAM}.

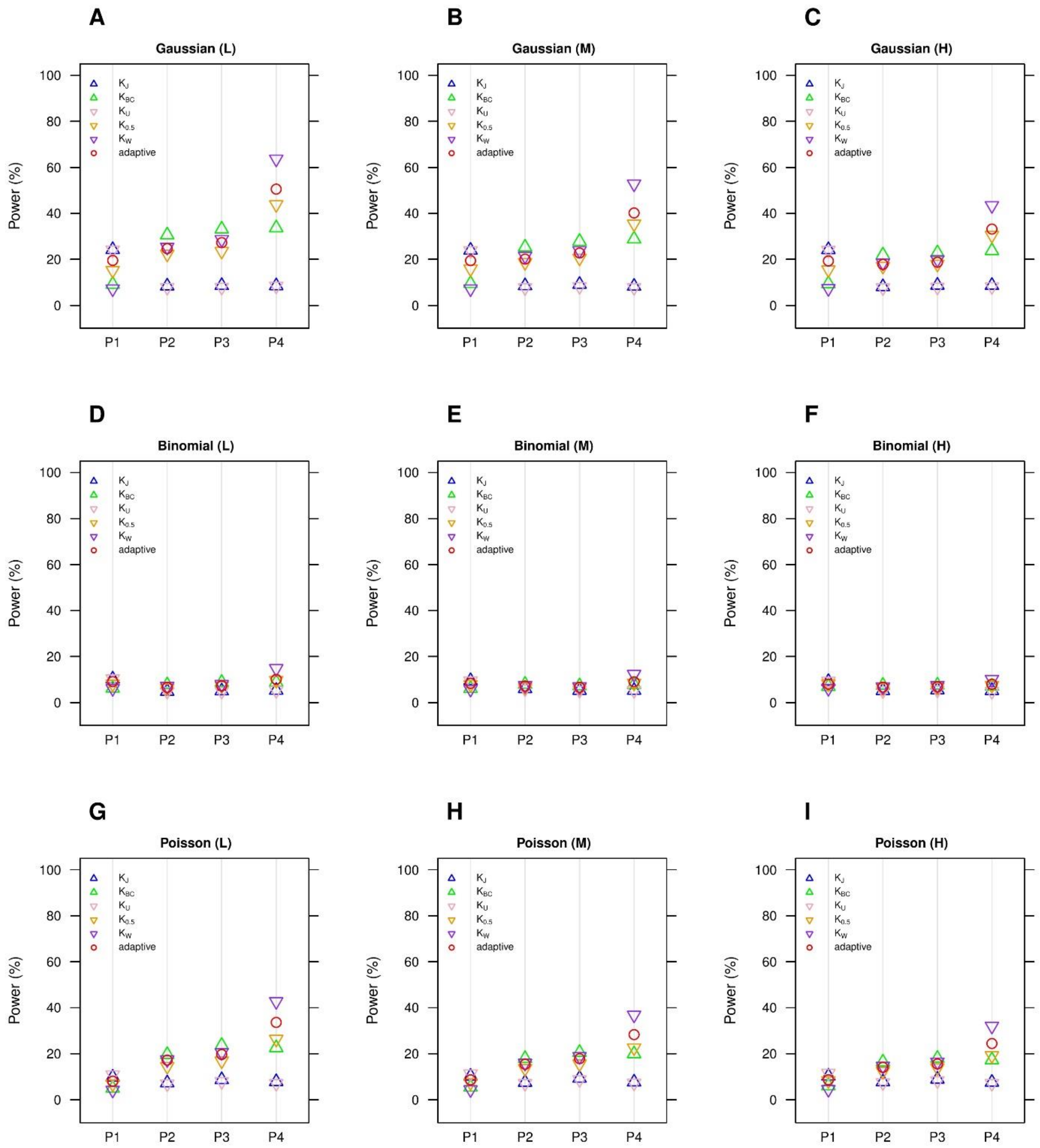

Figure S2. Estimated statistical powers for GLMM-MiRKAT/aGLMM-MiRKAT based on the random slope model with Gaussian, Binomial or Poisson responses (*n*=20) (Unit: %). L: low within-cluster correlation ($\rho_{j \neq j'}$ = $1/3$); **M:** medium within-cluster correlation ($\rho_{j \neq j'}$ = $1/2$); **H:** high within-cluster correlation ($\rho_{j \neq j'}$ = $3/5$).$\boldsymbol{K_J}$: Jaccard dissimilarity; $\boldsymbol{K_{BC}}$: Bray-Curtis dissimilarity; $\boldsymbol{K_U}$: Unweighted UniFrac distance; $\boldsymbol{K_{0.5}}$: Generalized UniFrac distance (θ=0.5); $\boldsymbol{K_W}$ **:** Weighted UniFrac distance; ***adaptive*****:** adaptive GLMM-MiRKAT (aGLMM-MiRKAT). **P1**, **P2**, **P3** and **P4** represent the four different association scenarios: **P1.** $\mathcal{A}$ = {50 random OTUs in lower half of abundance}; **P2.** $\mathcal{A}$ = {50 random OTUs}; **P3.** $\mathcal{A}$ = {50 random OTUs in upper half of abundance}; **P4.** $\mathcal{A}$ = {A random cluster among 10 clusters partitioned by PAM}.

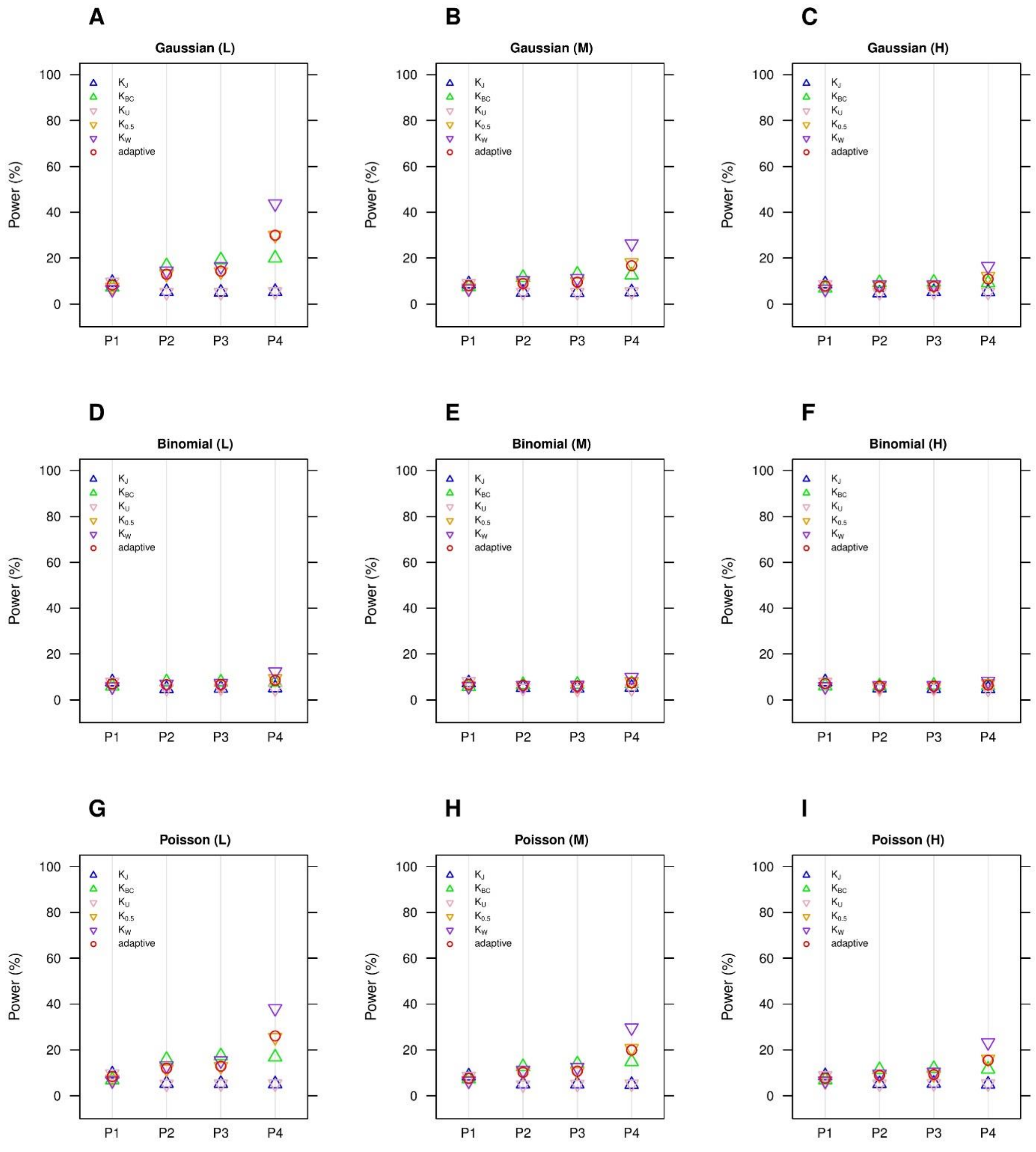

Supplement: Supplementary file 1 [file Data_Sheet_1.PDF]
